# Hot and Cold HCC: Uncoupling Viral Oncogenesis and Therapy

**DOI:** 10.3390/v17091255

**Published:** 2025-09-17

**Authors:** Laura Sneller, Keshav Mathur, Shyam Kottilil, Poonam Mathur

**Affiliations:** 1 College of Medicine, Drexel University College of Medicine, 2900 W Queen Ln, Philadelphia, PA 19116, USA; 2 Milken Institute School of Public Health, George Washington University, 950 New Hampshire Ave NW, Washington, DC 20001, USA; 3 Institute of Human Virology, University of Maryland School of Medicine, 725 W Lombard St., Baltimore, MD 21201, USA; skottilil@ihv.umaryland.edu

**Keywords:** hepatocellular carcinoma, treatment, immune checkpoint inhibitors, hot and cold tumors

## Abstract

Hepatocellular carcinoma (HCC) is rising in incidence globally. It is the sixth most common cancer and the third leading cause of cancer-related mortality worldwide. Infection with hepatitis B and/or C virus is a significant risk factor for developing HCC. These viruses exert their carcinogenicity in both direct and indirect ways, including induction of immune exhaustion with prolonged antigen exposure. Therefore, the best therapeutic option for HCC is prevention, i.e., Hepatitis B vaccination and treatment of viral hepatitis. However, when HCC develops because of viral hepatitis or other etiologies, long-lasting effects on the immune system remain even after viral suppression, which affect the response to HCC therapy. Recent studies have suggested a “hot” and “cold” model for HCC, in which the two kinds of HCC tumors have very distinct tumor microenvironments. The microenvironment for hot HCC makes these tumors amenable to immunotherapy with checkpoint inhibitors. Therefore, converting cold HCC tumors to hot tumors may make them susceptible to immunotherapy. In this review, we provide an overview of HCC epidemiology and prevention, an overview of tumor microenvironments of hot and cold HCC, the proposed mechanisms for converting cold tumors to hot tumors, and a concise summary of the evidence for combination checkpoint inhibitor therapy for HCC.

## 1. Introduction

Hepatocellular carcinoma (HCC) is the most common primary malignancy of the liver and a leading cause of cancer-related mortality worldwide [1]. HCC is noted even in ancient Egyptian papyri and Greco-Roman texts, but the understanding of disease pathogenesis was not until the 19th century when advances in microscopes allowed doctors to see abnormal cells in the liver [2]. HCC most frequently arises in the setting of chronic liver disease, particularly chronic hepatitis B virus (HBV) (primarily due to genomic instability due to HBV DNA integration) and hepatitis C virus (HCV) infections, though metabolic dysfunction-associated fatty liver disease (MAFLD), alcohol-related liver injury, and type 2 diabetes mellitus (T2DM) are increasingly recognized as major contributors to HCC development [1,3,4].

The pathogenesis of HCC involves a complex interplay of persistent inflammation, aberrant signaling, endoplasmic reticulum (ER) stress, and metabolic and genetic alterations [5]. Inflammatory stimuli such as IL-1B, IL-18, and TNF-a, along with activation of the NLRP3 inflammasome and chronic endoplasmic reticulum (ER) stress, promote hepatocyte damage and tumor progression [6]. Additionally, oncogenic pathways including PI3K/AKT/mTOR and Wnt/B-catenin are frequently dysregulated in HCC, through mutations in TERN, CTNNB1, and TP53, contributing to tumorigenesis and resistance to treatment [7,8,9].

The liver’s immune landscape is inherently tolerogenic by its constant exposure to gut-derived antigens. However, in chronic liver disease, this immune tolerance shifts toward a suppressive tumor microenvironment (TME), characterized by migration of regulatory T cells, myeloid-derived suppressor cells, tumor-associated macrophages, and increased expression of immune-checkpoint molecules such as PD-L1 and CTLA-4 [10,11,12]. These immunosuppressive forces collectively inhibit effective antitumor responses, allowing HCC to evade immune surveillance.

TME also provides the immunological context responsible for the dichotomy of “hot” versus “cold” tumors. Hot tumors are marked by high T-cell infiltration, PD-L1 expression, and an inflamed microenvironment. These tumors generally respond well to immune-checkpoint inhibitors (ICIs) [13,14]. In contrast, cold tumors exhibit immune exclusion, low PD-L1 expression, and minimal T-cell infiltration, leading to poor responses to immunotherapy [15,16]. Most HCCs fall along a spectrum between these two extremes, with a predominance of cold or immune-excluded phenotype [17].

Emerging treatment strategies aim to convert cold tumors into hot tumors by modulating TME and enhancing immune infiltration. Approaches include radiotherapy, oncolytic viruses, toll-like receptor (TLR) agonists, STING pathway activators, cytokine therapies, and combinations of ICIs with anti-angiogenic agents or epigenetic modulators [15,18,19,20]. These novel therapies seek to overcome immune resistance and improve therapeutic outcomes in HCC, underscoring the importance of immune classification in guiding treatment selection.

In this review, we aim to summarize the immunobiology of HCC and describe treatment in the context of hot versus cold tumor paradigms. We explore the mechanisms underlying immune exclusion in HCC, review therapeutic strategies aimed at reshaping the TME, and evaluate current and emerging approaches that seek to improve immunotherapy responses for HCC.

## 2. Epidemiology, Prevention, Screening, and Surveillance

HCC is the sixth most frequently diagnosed cancer worldwide and third leading cause of cancer mortality [15]. Its prevalence is high in sub-Saharan Africa, Southeast Asia, and East Asia, where both endemicity for chronic HBV and HCV infection is high. Males are more impacted by HCC than females, whose incidence is two to three-fold greater due to sex-specific determinants such as alcohol use and prevalence of smoking [16]. In the United States, HCC has increased threefold since the 1980s, primarily as a result of increased prevalence of chronic HCV infection and metabolic risk factors, including diabetes and obesity. Despite improvements in screening and early detection, the five-year survival rate for HCC is still very low, at about 20%.

Since chronic HBV infection is one of the most common risk factors for HCC, the main prevention strategy against HCC is universal HBV vaccination and effective treatment of chronic HBV and HCV infections [21]. Studies have shown that patients treated with anti-viral medications and sustained virological response have a reduced risk of HCC development [22]. In addition, the burden of HCC among patients with MAFLD is increasing, and, notably, up to one-third of MAFLD-related HCC cases occur in the absence of cirrhosis [23]. Current guidelines do not clearly address surveillance in non-cirrhotic MAFLD, but risk factors such as alcohol use, elevated FIB-4 and ALT, obesity, and diabetes are independently associated with HCC risk in patients with MAFLD [24]. While several risk score systems have been proposed, future validated tools are needed to guide surveillance decisions in this growing high-risk group [25]. Therefore, the avoidance of alcohol and tobacco use and maintenance of a healthy body weight are other strategies to avoid development of or progression of cirrhosis, and, ultimately, the risk of HCC [22].

HCC surveillance is recommended every six months in high-risk patients—such as those with cirrhosis of any etiology or chronic HBV infection—using ultrasound in combination with alpha-fetoprotein (AFP), which increases sensitivity for early detection from 45% to 63% [2]. HCC surveillance reduces mortality by ~50% and facilitates earlier tumor detection and higher rates of curative treatment (OR 2.24, 95% CI 1.99–2.52) [3,26,27]. Despite its benefits, hepatocellular carcinoma (HCC) surveillance remains underutilized, with fewer than 20% of patients with cirrhosis undergoing regular surveillance; even among those receiving hepatology care, nearly one-third receive inconsistent surveillance [27]. Surveillance underuse stems from multiple failures: missed diagnoses of liver disease or cirrhosis, provider failure to order surveillance (which is the most common cause), and patient nonadherence [27]. Barriers identified by primary care providers to order surveillance testing include lack of guideline knowledge, limited clinic time, and competing priorities [28]. Patients may also face logistical challenges with scheduling, test costs, and transportation [29]. Interventions using electronic health record reminders modestly improved surveillance rates—from 18.2% to 27.6% in one VA study [30]. However, long-term adherence remained low: only 23.3% of outreach/navigation patients received consistent surveillance over 18 months [31]. These findings highlight the need for more intensive, system-level interventions.

## 3. Immunopathogenesis

The direct cause of malignant transformation from chronic hepatitis infection into HCC is still not well known but is likely a result of persistent hepatic necro-inflammation. In HBV, oncogenesis mainly occurs as a result of viral genome into the host genome, which may increase its mutagenicity in the TERT promoter or p53 suppressor gene [1]. There is evidence to suggest that immune-mediated hepatocellular injury by HBV causes chronic necro-inflammation, which is highly pathogenic for HCC [32]. Chronic inflammation from chronic HCV infection also causes HCC development. HCV-associated HCC usually occurs in patients with cirrhosis or advanced fibrosis [1]. The key difference between HBV- and HCV-induced HCC is that HCV cannot insert itself into the host genome as HBV does [33]. A majority of the carcinogenic effects of HCV are through the action of its viral proteins [34].

**Figure 1:**
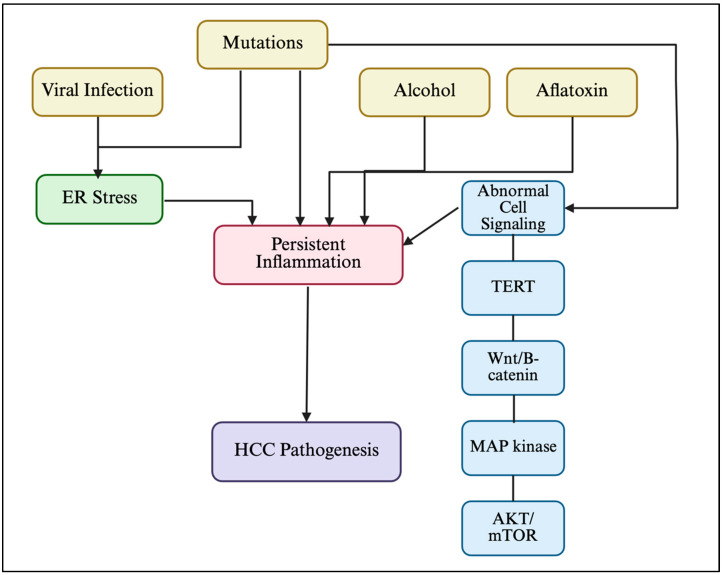
Summary of immunopathogenesis in hepatocellular carcinoma (HCC). ER= endoplasmic reticulum. Adapted from Kouroumalis et al. [33]. Created in biorender.com.

In non-viral etiologies for HCC, the primary mechanisms that contribute to HCC pathogenesis are persistent liver inflammation, abnormal cell signaling, and ER stress [34]. Inflammation is a key driver in HCC development, initiated by hepatocyte death that triggers immune responses via factors like HMGB1 and HDGF [35]. Several other pathways contribute to chronic inflammation and promote oncogenesis, which are summarized in Table 1 and Figure 1.

1. Activation of the NLRP3 inflammasome—through mitochondrial ATP release and lysosomal disruption—leads to caspase-1–mediated pyroptosis and the secretion of IL-1β and IL-18, further amplifying inflammation [36].
2. Chronic ER stress and UPR activation promote inflammation through pathways like NF-κB and TNF, contributing to HCC development across all tumor stages [5,6,26].
3. Abnormal activation of the PI3K/AKT/mTOR pathway promotes HCC by regulating key glycolytic enzymes, influencing proliferation, apoptosis, and metabolism [8,9].
4. Deregulated Wnt/β-catenin signaling, often due to CTNNB1 and AXIN1 mutations, cooperates with other oncogenes to drive HCC and mediate resistance to therapies [7,9].
5. miRNAs modulate HCC tumorigenesis by acting as oncogenes or tumor suppressors, affecting apoptosis and pathways like PI3K/AKT/mTOR and Wnt/β-catenin, and influencing initiation, progression, metastasis, and recurrence [37,38].

Other mechanisms of pathogenesis for HCC are comorbidity specific:

1. MAFLD and obesity key drivers include mutations in TERT, CTNNB1, ACVR2A, and the PNPLA3 I148M variant, as well as CCRK activation by obesity-related inflammation [3,39]. Additional mechanisms involve oxidative stress from fatty acid overload and iron deposition, which promote mitochondrial dysfunction and activate Wnt/β-catenin signaling, contributing to carcinogenesis [40].
2. Type 2 diabetes mellitus increases the risk of HCC and its recurrence independently of other factors like obesity, alcohol use, or cirrhosis [4,41]. Insulin resistance activates IGF-1 and IRS-1 signaling—particularly the PI3K/AKT/mTOR pathway—while metabolic dysregulation and upregulation of LINC01572 further promote HCC through enhanced glycolysis, ROS production, and p53 mutations [42,43].
3. Alcohol-related HCC is driven by ethanol metabolism into acetaldehyde, which causes oxidative stress and ROS accumulation, promoting DNA damage and carcinogenesis [44]. Additional mechanisms include gut-derived endotoxin-induced inflammation via TLR4 activation, IL-1β-mediated inflammasome signaling, and genetic variants in ADH, ALDH, PNPLA3, TM6SF2, and MBOAT7 that enhance susceptibility to ALD and HCC [45,46,47,48].

As summarized above, HCC arises from a complex interplay of viral, metabolic, inflammatory, and genetic factors. While chronic HBV and HCV infections remain major etiological drivers, other contributors—such as MAFLD, T2DM, and alcohol use—also significantly elevate risk. Central to HCC pathogenesis are persistent inflammation, aberrant signaling pathways like PI3K/AKT/mTOR and Wnt/β-catenin, endoplasmic reticulum stress, and the dysregulation of non-coding RNAs. Genetic variants and metabolic disturbances further modulate susceptibility and tumor progression.

## 4. Tumor Microenvironment

The liver’s microenvironment contains a large reservoir of immune cells and maintains homeostasis by being highly tolerogenic to gut bacterial metabolites [11,49]. This tolerance is established through the interaction between liver-resident cells and peripheral leukocytes. However, chronic liver diseases drive inflammatory dysregulation and necro-inflammation, compromising this tolerance, and, ultimately, exploit the liver’s immune tolerance to contribute to HCC pathogenesis [49]. For example, the liver’s unique immune tolerance is maintained by non-parenchymal cells, including Kupffer cells, hepatic stellate cells (HSCs), and liver sinusoidal endothelial cells (LSECs). These cells have immunosuppressive mechanisms which can inadvertently contribute to HCC pathogenesis [50]. Kupffer cells promote immune escape via IL-10 secretion, PD-L1 upregulation, co-stimulatory molecule downregulation (CD80/CD86), IDO production, and the recruitment of Treg and Th17 cells, ultimately inducing T-cell exhaustion through PD-1/PD-L1 interaction [11,51,52,53]. HSCs facilitate MDSC and Treg accumulation via HGF secretion and promote T-cell apoptosis through PD-L1 expression [54]. LSECs also induce Treg activation via TGF-β and express PD-L1. Additionally, tumor-associated fibroblasts contribute to immune suppression by impairing NK-cell function (via PGE2 and IDO) and promoting MDSC recruitment through IL-16 and CXCL12 secretions [55].

The majority of HCCs arise within a chronically inflamed, immunosuppressive microenvironment characterized by diminished T-cell co-stimulatory signaling and increased expression of immune-checkpoint molecules, resulting in impaired T-cell effector function [12]. The tumor microenvironment facilitates tumor progression by providing a sanctuary for malignant cells to evade immune system surveillance. HCCs exhibit an intermediate mutational load and employ multiple evasion mechanisms to sustain growth [17]. Recent single cell RNA sequencing analyses have demonstrated substantial inter-patient heterogeneity among HCC tumor cells, whereas TME displays more conserved gene expression signatures among individuals [56] These findings underscore the potential of therapeutic strategies targeting the tumor microenvironment to overcome tumor-associated immune resistance in HCC.

Immune system evasion is partly mediated by immunosuppressive cell populations within the tumor microenvironment, including myeloid-derived suppressor cells (MDSCs), regulatory T cells (Tregs), and tumor-associated macrophages (TAMs) [10,57]. MDSCs suppress cytotoxic T- and NK-cell activity through cytokine-mediated recruitment (e.g., IL-6, CCL26, VEGF), metabolic disruption (arginase, ROS, NOS2), and checkpoint ligand expression (Galectin-9, PD-L1) [11,54,55,58]. Tregs promote immune evasion by suppressing CD8+ T-cell cytotoxicity, inhibiting pro-inflammatory cytokines (TNF-α, IFN-γ), and secreting IL-10, TGF-β, and IL-35, with recruitment driven by CCR6/CCL20 signaling and further regulated by lncRNAs and EGFR pathways [59,60,61,62,63,64,65]. TAMs, derived from monocytes, exhibit M1 (pro-inflammatory) or M2 (immunosuppressive) polarization, with M2 TAMs producing IL-10, TGF-β, VEGF, and IDO, contributing to Treg recruitment, NK-cell suppression, and tumor progression [66,67,68]. Additional immunosuppressive cells, including PD-1+ B cells, Th17, Th2, tumor-associated neutrophils, fibroblasts, and CTLA-4+ dendritic cells, further exacerbate immune evasion and correlate with poor prognosis in HCC [69,70]. Tumor-intrinsic pathways in HCC also modulate immune escape: CTNNB1 mutations and WNT-β-catenin activation suppress CCL5 and DC recruitment, impair NKG2D ligand expression on HCC cells, and reduce NK-cell cytotoxicity [71]; p53 loss and CDK20 activation increase MDSC infiltration [72]; MYC overexpression, seen in 50–70% of cases, upregulates PD-L1 [73]; and chronic HBV infection elevates PD-L1 on Kupffer cells, leukocytes, and LSECs, enhancing immune suppression [12].

Immune checkpoints—such as PD-1, CTLA-4, LAG3, and TIM3—also play central roles in mediating T-cell exhaustion and promoting immune evasion by interacting with ligands expressed on tumor and stromal cells [10,58,69,74]. CTLA-4 inhibits T-cell proliferation and enhances Treg activity, while PD-1, expressed on multiple immune cells, suppresses antigen-specific T-cell responses upon binding PD-L1, which is upregulated in tumor and stromal cells and correlates with poor prognosis [12,69,74,75,76]. LAG3 and TIM3 are highly expressed on TILs and contribute to T-cell dysfunction via MHC-II and galectin-9 interactions, respectively, further exacerbating immune suppression in HCC [77,78]. Combination strategies targeting PD-1, LAG3, and TIM3 are under investigation to overcome resistance to immune-checkpoint blockade [79,80,81].

Cytokines such as TGF-β, IL-10, and VEGF contribute to immunosuppression by modulating immune cells [11]; TGF-β, produced by tumor cells, TAMs, and Tregs, drives M2 macrophage polarization and Treg expansion, inhibits CD8+ T and NK-cell function, impairs DC activation, and correlates with poor responses to sorafenib and pembrolizumab [29,82]; IL-10, secreted by tumor and immune cells, reduces T-cell infiltration, upregulates PD-L1, increases MDSCs, and predicts poor prognosis [83,84]; VEGF promotes angiogenesis, impairs APC and T-cell function, expands MDSCs and Tregs, and elevates PD-1/PD-L1 expression, and its 6p21 amplification fosters immune escape [85]; thus, targeting these cytokines represents a potential strategy to counter HCC-associated immunosuppression.

The main etiologies of HCC use varied methods for pathogenesis. Chronic antigen presentation invoked by HBV induces T-cell exhaustion and immunosuppression [86]. HCV evades immunity through high mutation rates and interference with DNA sensing mechanisms [87]. Alcohol-related liver disease and MAFLD trigger sterile inflammation, which activates macrophages, monocytes, and neutrophils [88]. Lastly, bacterial extracts from MAFLD-HCC patients have been found to suppress cytotoxic T-cell responses ex vivo [89].

**Figure 2:**
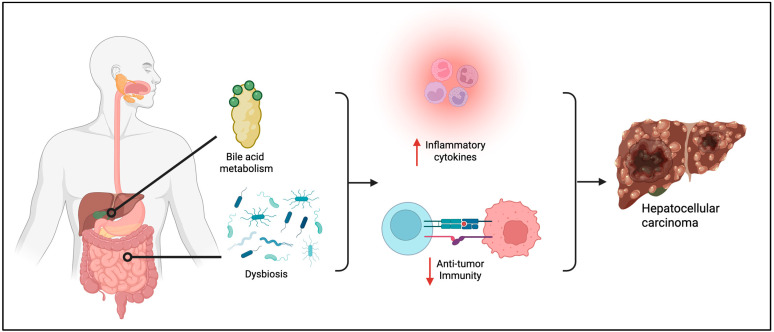
Gastrointestinal dysbiosis and bile acid metabolism contributing to the development of hepatocellular carcinoma. Created in biorender.com.

The liver microbiome is usually highly tolerogenic to the gut microbiome and bile acid metabolism, resulting in expansion of regulatory T cells induced by IL-10 from Kupffer cells [11,49,51,90,91]. However, disruption in the homeostasis of either bile acid metabolism and/or the microbiome can contribute to HCC development through immune activation and the release of inflammatory cytokines; for example, bacterial translocation in MAFLD patients can activate toll-like receptor 4 (TLR4) via LPS, resulting in inflammatory cytokine production and acceleration of tumor progression [92]. Dysregulation of the microbiome can contribute to the progression of liver disease to HCC not only by increased intestinal permeability that drives infections (since patients with HCC have a microbiome characterized by more pathogenic than beneficial bacteria) but also by hindering energy supply (by a decrease in butyrate-producing bacteria) and interfering with normal cellular physiologic functions and antitumor immunity (via promotion of a senescence-associated secretory phenotype of hepatic stellate cells) [91,93] (Figure 2). It is possible that characterizing the gut microbiome prior to the use of immunotherapy for HCC can predict clinical response [91]. For example, one study found that HCC patients with higher abundance of *Lachnospiraceae bacterium-GAM79* and *Alistipes sp Marseille-P5997* in fecal samples had longer progression-free survival and overall survival rates with anti-PD-1 therapy than those patients with lower abundance of the bacteria [94]. Another study found that patients with *Lachnoclostridium, Lachnospiraceae,* and *Veillonella* in abundance were in remission before immunotherapy for HCC, whereas *Prevotella 9* was significantly abundant in patients with progressive disease. After immunotherapy, patients with abundance of both *Lachnoclostridium* and *Prevotella 9* had better overall and progression-free survival than the other patients [95]. Using the microbiome as a potential therapeutic target to modulate cancer immunotherapy response remains a developing area of investigation.

Overall, the immunosuppressive microenvironment of HCC arises from a complex interplay between chronic inflammation, the gut microbiome, liver-resident and infiltrating immune cells, cytokines, immune checkpoints, tumor-associated stromal cells, and tumor-intrinsic genetic alterations. This multifaceted network fosters immune evasion by impairing cytotoxic T-cell and NK-cell responses, expanding regulatory and suppressive cell populations, and upregulating inhibitory pathways such as PD-1, CTLA-4, LAG3, and TIM3. Understanding these interconnected mechanisms not only elucidates HCC pathogenesis but also highlights multiple therapeutic targets within the tumor microenvironment that may enhance the efficacy of emerging immunotherapy.

## 5. Hot and Cold HCC Tumors

The classification of “hot” and “cold” tumors encompasses characteristics of cancer cells, including the microenvironment of the tumor, immune modulators present, and the signaling mechanisms utilized [96]. The TME plays a significant role in determining which tumors are cold and hot, due to the interactions between tumor cells and their surrounding stroma. Specifically, the type of immunosuppression within the TME drives the hot and cold phenotype: intrinsic immunosuppression due to genetic alterations and oncogenic pathway activation leads to cold tumors, and locally adaptive immunosuppression, which promotes T-cell infiltration, leads to hot tumors [13].

**Figure 3:**
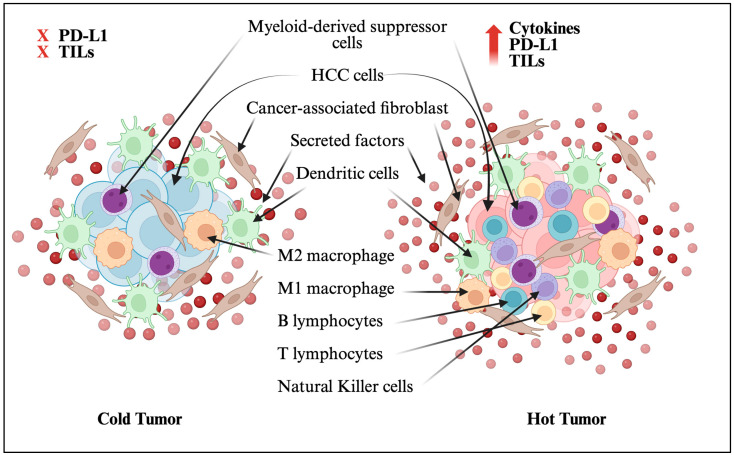
Hot and cold HCC tumor microenvironments. Adapted from Wang et al. [96]. Created in biorender.com.

Cold and hot tumor microenvironments (TMEs) are distinguished by differing immune cell infiltration and cytokine profiles (Figure 3). Hot tumors are marked by abundant TILs, PD-L1 overexpression, genomic instability, and preexisting antitumor immunity, whereas cold tumors lack these features [13,14]. Hot tumors are also characterized by activation of immune-stimulatory pathways such as CD28/B7 and CD40/CD40L; robust infiltration by T cells, NK cells, and dendritic cells (DCs); lower levels of suppressive cytokines; and strong antitumor immunity. In this setting, immune activation factors secreted by tumor cells promote immune cell proliferation, and PD-L1-expressing tumor cells are targeted for destruction [97]. Conversely, cold tumors exhibit reduced populations of T cells and NK cells due to activation of immunosuppressive immune-checkpoint pathways, such as PD-1/PD-L1 and CTLA-4, along with elevated levels of immunosuppressive cytokines like IL-10 and TGF-β, collectively inhibiting immune cell proliferation and activation. Moreover, oncogenic drivers (including hypoxia) cause the expression of HIF-1a, which promotes a cold TME via increased PD-L1 expression [98]. This has been shown by in vitro data in which the inhibition of HIF-1a changes the TME to hot, reactivating tumor-infiltrating lymphocytes and makes immunotherapy more effective [98].

## 6. Turning Cold HCC into Hot HCC

Early-stage HCC can be treated with surgical resection or liver transplantation. For unresectable HCC, orthotopic liver transplantation—often preceded by bridging therapies such as chemotherapy, transarterial chemoembolization, or percutaneous ablation—offers an effective surgical alternative [99,100].

The use of immunotherapy to treat HCC has increased in the last decade. Although multiple molecular pathways contribute to HCC pathogenesis, targeted therapies have had limited clinical success. Sorafenib, a multikinase inhibitor targeting Raf-1, B-Raf, VEGFR-1/2/3, and PDGFR-β, remains the most extensively studied agent [101,102]. In the SHARP trial, sorafenib significantly improved overall survival (10.7 vs. 7.9 months; HR 0.69; *p* < 0.001), despite a low objective response rate (partial response in 2%) in 602 patients with Child–Pugh A cirrhosis compared to placebo [103]. Combining sorafenib with chemotherapy, however, has produced mixed outcomes: a phase II trial showed improved survival when used with doxorubicin, but combinations with other targeted agents—such as sunitinib, cetuximab, erlotinib, and everolimus—either failed to outperform sorafenib or led to increased toxicity [104,105].

**Figure 4:**
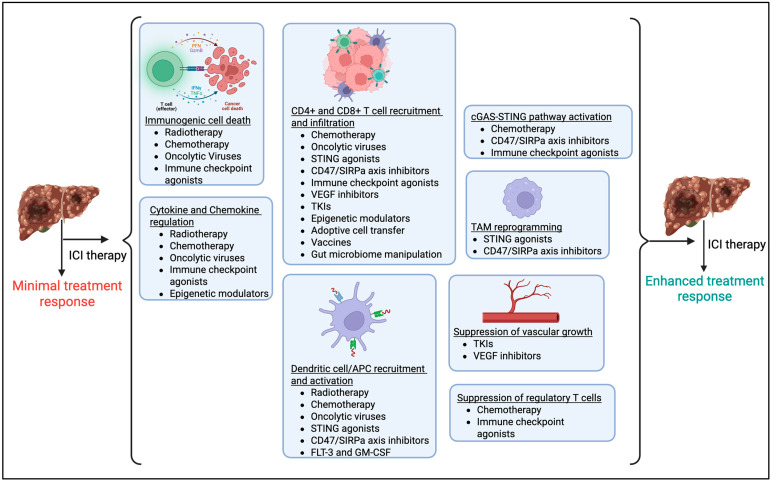
Mechanisms of action of combination therapy. ICI = immune checkpoint inhibitor. TAM= tumor-associated macrophages. APC = antigen-presenting cell. TKIs = tyrosine kinase inhibitors. VEGF = vascular endothelial growth factor. Created in biorender.com.

Treatment failure with sorafenib and other immunotherapies is likely due to the presence of cold HCC. Since immunotherapy with checkpoint inhibitors is effective against hot tumors, modulating the TME to enhance immune infiltration and convert “cold” into “hot” tumors could be effective for improving treatment outcomes [96]. Specific strategies to convert “cold” to “hot” HCC to enhance the response immune-checkpoint inhibitors (ICIs) are summarized below and in Figure 4.

### 6.1. ICIs and TKIs

**Figure 5:**
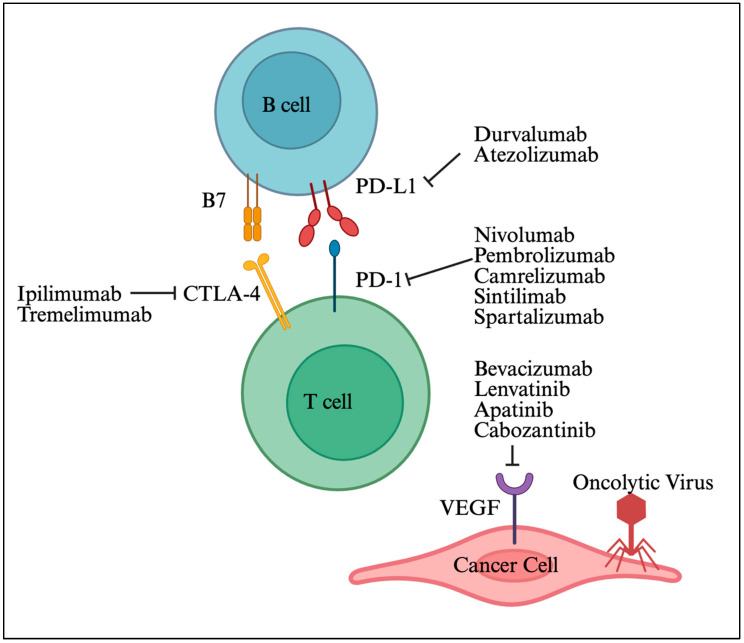
HCC treatment targets. Adapted from Yu et al. [106]. Created in biorender.com.

Combining ICIs with anti-angiogenic agents or TKIs has emerged as a leading therapeutic strategy for advanced HCC [106]. The FDA-approved atezolizumab (anti-PD-L1) plus bevacizumab (anti-VEGF) demonstrated superior efficacy over sorafenib in the IMbrave150 trial (ORR 33% vs. 13%, median OS 19.2 vs. 13.4 months) [107]. The MORPHEUS-liver study added tiragolumab (anti-TIGIT) to this regimen, showing improved ORR (42.5% vs. 11.1%) and PFS (11.1 vs. 4.2 months) [108]. Lenvatinib plus pembrolizumab also showed promising efficacy (ORR 36–46%, OS ~22 months) [107]. Camrelizumab with apatinib (anti-VEGFR2) reported ORRs of 34.3% (first line) and 22.5% (second line) [109]. STRIDE (tremelimumab + durvalumab) improved survival (OS 16.4 months) versus sorafenib (13.8 months) [110]. Dual ICI (nivolumab + ipilimumab) achieved an ORR of 32% and OS of 22.8 months in CheckMate-040 [111]. Trials targeting emerging checkpoints (e.g., LAG-3, TIM-3) in combination with PD-1/PD-L1 are underway (NCT05337137, NCT03099109). Locoregional therapies like Y90 radioembolization or stereotactic body radiotherapy (SBRT) combined with ICIs have shown ORRs up to 57% [112]. In one study, chemotherapy plus ICI (e.g., FOLFOX4 + camrelizumab) yielded an ORR of 29.4% [113]. Common ICIs used in the treatment of HCC are shown in Figure 5.

### 6.2. Radiotherapy

Radiotherapy has emerged as a promising adjunct to ICIs due to its immunostimulatory effects. By inducing immunogenic cell death (ICD), radiotherapy triggers the release of damage-associated molecular patterns (DAMPs), including high mobility group protein 1 (HMGB1), calreticulin (CRT), heat shock proteins, and ATP. Radiotherapy also enhances major histocompatibility complex class I (MHC-I) expression, improving antigen presentation to CD8+ T cells [114,115]. Ultimately, these processes activate the immune system, making HCC tumors more “hot” and susceptible to immunotherapies, especially ICIs. Additionally, radiotherapy activates type I interferons (IFNs) via the cGAS-STING pathway in DCs, further promoting cross-priming and tumor regression [114]. It is possible that radiation upregulates immune-checkpoint molecules such as PD-L1, which contribute to adaptive resistance; this highlights the rationale for combining radiotherapy with PD-1/PD-L1 inhibitors [114,116,117].

### 6.3. Chemotherapy

Although traditionally considered immunosuppressive, chemotherapy has the potential to enhance antitumor immunity in HCC by inducing ICD, releasing neoantigens, and activating Type I IFN signaling that promotes antigen-presenting cell (APC) recruitment [118]. Platinum-based agents such as cisplatin upregulate MHC-I expression and co-stimulatory molecules (CD70, CD80, CD86), enhancing CD8+ T-cell activation [119,120]. Additionally, agents like paclitaxel activate cGAS-STING pathways, further boosting pro-inflammatory responses [121]. Locoregional approaches, including transarterial chemoembolization (TACE), deliver high-dose chemotherapy directly to tumors, inducing ICD and modulating immune cell populations (i.e., increasing CD4+/CD8+ ratios and reducing regulatory T cells) [122].

### 6.4. Oncolytic Viruses (OVs)

OVs represent a novel therapeutic class with both direct tumor-lysing and potent immune-stimulating properties, selectively infecting and eliminating cancer cells while sparing normal tissues [123]. Through selective replication in tumor cells, OVs induce ICD, release tumor antigens and danger signals, and recruit DCs and innate lymphoid cells, which enhances antigen presentation and initiates both innate and adaptive immune responses [124]. OVs have been shown to directly convert “cold” tumors into “hot” phenotypes via increased immune cell infiltration and cytokine production, which result in tumor cell lysis and provoke a systemic antitumor immune response [116,124]. JX-594 is the most extensively studied OV in HCC. It directly lyses tumor cells while expressing GM-CSF to boost antitumor immunity, demonstrating promising activity in early clinical trials [125].

### 6.5. STING (Stimulator of IFN Genes) Pathway

The STING pathway plays a pivotal role in innate immunity by sensing cytosolic DNA and inducing Type I IFN responses that enhance antigen presentation and T-cell priming [126]. In HCC, STING agonists activate DCs, reprogram TAMs, and increase CD8+ T-cell infiltration via chemokine upregulation such as CXCL9 and CXCL10 [126]. Furthermore, STING activation upregulates immune-checkpoint molecules like PD-L1, promoting adaptive immune resistance. Therefore, STING agonists can be used in combination with ICIs, since they convert cold HCC into hot HCC by release of Type I IFNs, recruitment and activation of immune cells, enhancement of T-cell activity via CD8+ T-cell infiltration, and reprogramming of macrophages [126]. The oral STING agonist MSA-2 has shown systemic antitumor efficacy and synergy with PD-1 blockade in preclinical models [19].

### 6.6. CD47-SIRPa Axis

The CD47-SIRPa axis promotes immune evasion in HCC and a cold HCC phenotype since overexpression of CD47 allows HCC tumor cells to transmit inhibitory signals upon binding with SIRPa. Therefore, blocking this axis activates the STING pathway, restores phagocytosis in macrophages and DCs, promotes antigen cross presentation, and enhances recruitment of cytotoxic immune cells (i.e., CD8+ T cells), reprogramming the TME and converting the HCC tumor into a hot phenotype that works synergistically with ICIs, chemotherapies, and radiotherapies [123,127]. Novel agents like SIRPa-Fc fusion proteins and GPC3-CD47 bispecific antibodies have demonstrated potential to convert “cold” HCC tumors into “hot” immune-responsive tumors, but their optimal integration with ICIs remains under investigation [128].

### 6.7. FLT-3L and GM-CSF

FMS-like tyrosine kinase 3 ligand (FLT-3L) and the granulocyte-macrophage colony-stimulating factor (GM-CSF) are important cytokines involved in DC development and function. These cytokines have exhibited emerging therapeutic roles for HCC, by primarily focusing on the recruitment, activation, and maturation of DCs to convert cold HCC into hot HCC. FLT-3L promotes DC proliferation and enhances antitumor immunity through in situ vaccination strategies, such as adenoviral vectors expressing FLT-3L, which have been shown to potentiate radiation therapy and induce Th1 immune response in HCC models [129]. GM-CSF also promotes DC and macrophage expansion and is a component of JX-594, as described above [125]. IL-2 can be combined with GM-CSF to enhance DC-mediated T-cell activation [130]. By enhancing DC development and function, FLT-3L and GM-CSF can reshape the TME to make cold HCC tumors more susceptible to ICIs.

### 6.8. Immune Checkpoint Agonists

Agonists targeting co-stimulatory immune-checkpoint receptors such as CD40 and OX40 have shown potential in increasing antitumor immunity in HCC to convert tumors from cold to hot phenotypes. These are an emerging class of agents that are under investigation to target the STING pathway, induce ICD, and promote CD8+ T-cell infiltration via several mechanisms. For example, CD40 is expressed on APCs and interacts with CD40L on activated T cells to promote DC maturation and IL-12 secretion, ultimately stimulating T-cell response. CD40 agonists improve DC activation, increase T-cell infiltration, and suppress tumor progression in preclinical HCC models [131]. Another strategy uses OX40 since it is expressed on activated T cells and binds to OX40L on APCs to amplify T-cell effector function and memory [132]. Combination strategies using OX40 agonists with TLR9 stimulation have resulted in enhanced CD4+ and CD8+ T-cell activity, suppression of regulatory T cells and myeloid-derived suppressor cells, and durable immune memory in HCC models [132].

### 6.9. VEGF (Vascular Endothelial Growth Factor) Inhibition

Tumor progression is supported both by immune evasion and aberrant vascularization, with hypoxia-induced VEGF playing a central role in neovascularization and tumor growth [133]. VEGF impairs DC maturation and promotes the expansion of immunosuppressive cells, while also facilitating immune escape by restricting CD8+ T-cell activity [134]. VEGF/VEGFR monoclonal antibodies (e.g., bevacizumab and ramucirumab) and TKIs (e.g., orafenib, sunitinib and L = lenvatinib) not only suppress tumor vascular growth but also enhance immune infiltration and responsiveness to immunotherapy in HCC [74,103,104,105,135,136]. Notably, lenvatinib improves the efficacy of PD-1 blockade by targeting the FGFR4-GSK3B axis, reducing regulatory T-cell differentiation and boosting T-cell-mediated cytotoxicity [137,138]. Thus, combining anti-angiogenic therapy with immunotherapy offers an effective strategy for HCC treatment.

### 6.10. Chemokine Regulation

HCC tumors evade the immune system through impaired chemokine signaling. Reduced expression of Th1-type chemokines, such as CXCL9 and CXCL10, contributes to poor CD8+ T-cell recruitment and limited responses to immunotherapy [139]. Epigenetic silencing by DNA methyltransferases can suppress chemokine gene expression (including CCL5) and further contributes to immune evasion [140]. In preclinical HCC models, epigenetic modulators, such as DNA demethylating agents and HDAC8 inhibitors, have been shown to restore chemokine expression and increase CD8+ T-cell infiltration [141]. Therefore, targeting chemokine regulation through epigenetic intervention may enhance immunotherapy efficacy in immune-excluded HCC.

### 6.11. Adoptive Cell Transfer (ACT)

ACT therapy, including chimeric antigen receptor T-cell (CAR-T) therapy, enhances tumor-specific immunity by engineering patient-derived immune cells for reinfusion [142,143]. While CAR-T therapy has shown success in hematologic malignancies, its application in solid tumors like HCC remains limited due to shared antigen expression with normal tissues [144]. Glypican-3 (GPC3) is a promising CAR-T target since it is highly expressed in HCC and absent in normal adult tissue [134]. Clinical trials have demonstrated that GPC3-targeted CAR-T cells, especially those co-expressing cytokines like IL-7 and CCL19, exhibit enhanced expansion and robust antitumor activity [145]. GPC3-specific CAR-modified Vd1 T cells have also shown improvement in cytotoxic responses in HCC [146]. Combining ACT with ICIs may improve efficacy, but this combination must be further explored in clinical trials since it introduces a heightened risk of immune-related toxicity.

### 6.12. Vaccines

Vaccine-based cancer therapy shows strong potential in enhancing immune recognition of tumors, particularly through neoantigen vaccines that target tumor-specific antigens with high immunogenicity [147]. In HCC, the immunosuppressive TME can limit vaccine efficacy, but combining vaccines with ICIs has shown synergistic antitumor effects [148,149,150]. Neoantigen-loaded DC vaccines have shown high specificity and minimal off-target effects, and novel platforms such as acidic/photosensitive DC nanovaccines have successfully converted “cold” to “hot” tumors [151]. Peptide-based vaccines targeting GPC3 and ASPH have also shown promise, particularly when fused with chemokines of viral platforms to increase cytotoxic T-cell infiltration and response to ICIs [148,152].

### 6.13. Manipulation of Gut Microbes

*Mycobacterium anthropophilum* and *Bifidobacterium bifidum* were found to exert regulatory function on ICIs in 2015 [91]. Since then, several clinical trials have demonstrated the enhanced efficacy of ICIs after microbiome manipulation in patients with HCC [91]. These interventions have included fecal microbiota transplantation, prebiotics (i.e., use of potato starch), and probiotics, i.e., administration of *Lactobacillus rhamnosus Probio-M9,* which enhance CD8 T-cell infiltration into tumors and efficacy of ICIs (PD-L1 and CTLA-4 inhibitors) [92]. The mechanisms for enhanced efficacy of ICIs vary based on the bacteria and immune cell they influence. Generally, bacteria that activate B cells enhance ICIs by regulating gut homeostasis and promoting antitumor immunity, macrophages and dendritic cells enhance antitumor effects, NK cells improve hepatic immunity and restore function, and CD8+ T cells strengthen antitumor immunity via enhanced IGNg and granzyme B production, memory T-cell differentiation [153]. A recent meta-analysis shows that ultimately, the relationship between the microbiome and ICI efficacy varies by microbial type, tumor type, treatment strategy, sampling site, and bacterial model configurations [154].

## 7. Assessing Cold to Hot HCC Conversion

Conversion of cold into hot HCC greatly alters the TME. Therefore, measurement of biomarkers before and after implementing the aforementioned therapies may be beneficial in assessing the tumors’ enhanced potential for response to ICI therapy [155]. In clinical practice, AFP is the biomarker commonly used for serial monitoring. An overall decline in AFP suggests a favorable response to treatment and a shift away from a cold phenotype, since AFP plays a role in HCC escaping immune surveillance by inhibiting lymphocyte and macrophage function and maturation and activation of DCs [156]. Use of other biomarkers in clinical practice is not yet standard-of-care, but theoretically, the biomarkers discussed below could be used to monitor treatment response, especially since most have been shown to be associated with HCC prognosis and immunotherapy efficacy. Biomarkers that have the potential to demonstrate successful cold to hot HCC conversion can be grouped into immune cell biomarkers, cytokine and signaling pathway biomarkers, and metabolic and genomic biomarkers.

Measuring immune cell biomarkers could assess the density of cells responsible for tumor lysis. Specifically, an increase in the density of TILs (i.e., CD8+ T cells) and CXCL9 and CXCL10, which activate T cells and IFNg secretion from effector T cells, would demonstrate conversion of an HCC tumor into the hot phenotype. Though measuring TIL density is not routinely used in clinical practice, it has been identified as an independent prognostic factor for overall survival in HCC [157]. CXCL9 and CXCL10 are also effective biomarkers of immunotherapy efficacy, but measurements of these have not been integrated into clinical practice [158]. An increase in the density of mature DCs could also symbolize increased antigen presentation and immune activation, as there is data that associates DC density with HCC prognosis; however, DC density is not yet used routinely in clinical practice [159]. High levels of Tregs have been correlated with poor outcomes in patients with HCC, so a decline in Tregs would indicate that the tumor has shifted to a hot phenotype. but its use as a prognostic marker is not yet clear or standardized for clinical practice [160].

Measurement of cytokine and signaling pathway biomarkers could also assess for a more pro-inflammatory TME or reduction in a regulatory/immunosuppressive TME, which would characterize hot-to-cold HCC conversion. For example, an increase in PD-L1 and CXCL12 and reduction in TGFB would suggest that these tumors would have a better response to ICI therapy. However, use of PD-L1 as a biomarker is a challenge clinically due to heterogeneity in tissue expression and technical challenges with isolation of the marker [161,162]. Lower levels of CXCL12 and higher levels of TGFB correlate with poor HCC prognosis, but more studies are needed to validate their clinical utility in monitoring HCC treatment response [163,164]. Conversely, lower serum levels of IFNg in HCC patients are associated with advanced tumor stage and worse prognosis, so monitoring for increases in serum IFNg is a potential modality for determining cold to hot HCC conversion, but this needs to be validated for standard clinical use [165]. Use of changes in the STING signaling pathway (which can be assessed using a PET tracer, [18F]FBTA), is under investigation to assess additional immune remodeling, but its feasibility in the clinical setting is challenging [166].

Lastly, the assessment of metabolic and genomic factors may guide prediction for ICI response. Reduction in HIF-1a may indicate a tumor more amenable to ICI therapy by altering PD-L1 expression [98]. In addition, assessment of the gut microbiome and bile acid metabolism may also provide insight into how the TME can be influenced to convert the tumor phenotype. The use of HIF-1a and changes in the microbiome and bile acid metabolism as markers of cold to hot HCC conversion is still under investigation.

## 8. Conclusions

HCC arises from a multifactorial interplay of viral, metabolic, genetic, and inflammatory factors, culminating in a chronically immunosuppressed TME that supports immune evasion and tumor progression [5,11,26]. Although immunotherapies such as ICIs have significantly advanced the therapeutic landscape, their efficacy in HCC remains limited by the predominance of “cold” or immune-excluded tumor phenotypes, which have poor immune cell infiltration and strong immunosuppressive cytokine production [17,167].

The emerging paradigm of “hot” versus “cold” tumors provide a valuable framework for understanding therapeutic response variability and designing combination strategies for treating HCC. Therapeutic strategies that aim to convert cold HCCs into hot tumors, hold promise for enhancing immune responsiveness [18,19,20,141]. Notably, the integration of these modalities into clinical practice is being supported by encouraging early-phase trial data and preclinical evidence.

Moving forward, a deeper understanding of HCC immune subtypes and development of reliable predictive biomarkers will be essential to guide personalized immunotherapy. By tailoring treatments based on tumor immunophenotype, clinicians may overcome immune resistance and achieve more durable and effective responses. Precision immuno-oncologic strategies have the potential to shift the therapeutic landscape and improve outcomes for patients with HCC.

## Figures and Tables

**Table 1:** Pathways that contribute to chronic inflammation and promote HCC oncogenesis.

Pathway/Insult	Effects that Promote Carcinogenesis
NLRP3 inflammasome	Caspase-1–mediated pyroptosis Secretions of IL-1β and IL-18
Chronic ER stress and UPR activation	NF-κB activation TNF production
PI3K/AKT/mTOR upregulation	Increase proliferation Reduced apoptosis Altered cellular metabolism
CTNNB1 and AXIN1 mutations	Deregulated Wnt/β-catenin signaling
Genetic mutations from MAFLD and obesity	CCRK activation Oxidative stress from fatty acid overload and iron deposition Mitochondrial dysfunction
miRNAs	Disruption of apoptosis pathways, influencing initiation, progression, metastasis, and recurrence
Type 2 Diabetes Mellitus (insulin resistance)	IGF-1 and IRS-1 signaling Enhanced glycolysis ROS production p53 mutation
Ethanol metabolism into acetaldehyde	DNA adduct formation Oxidative stress ROS accumulation TLR4 activation IL-1β-mediated inflammasome signaling Genetic variants in ADH, ALDH, PNPLA3, TM6SF2, and MBOAT7
